# Periodic instability and restoration of cratonic lithosphere

**DOI:** 10.1093/nsr/nwaf027

**Published:** 2025-01-23

**Authors:** Lijun Liu, Ling Chen, Zebin Cao, Xiaotao Yang, Andrea Stevens Goddard, Rixiang Zhu

**Affiliations:** State Key Laboratory of Lithospheric and Environmental Coevolution, Institute of Geology and Geophysics, Chinese Academy of Sciences, Beijing 100029, China; State Key Laboratory of Lithospheric and Environmental Coevolution, Institute of Geology and Geophysics, Chinese Academy of Sciences, Beijing 100029, China; State Key Laboratory of Lithospheric and Environmental Coevolution, Institute of Geology and Geophysics, Chinese Academy of Sciences, Beijing 100029, China; Department of Earth, Atmospheric, and Planetary Sciences, Purdue University, West Lafayette, IN 47907, USA; Department of Earth and Atmospheric Sciences, Indiana University, Bloomington, IN 47405, USA; State Key Laboratory of Lithospheric and Environmental Coevolution, Institute of Geology and Geophysics, Chinese Academy of Sciences, Beijing 100029, China

**Keywords:** dense craton root, lithospheric weakness, delamination and relamination, uplift and subsidence, periodic craton deformation, supercontinent cycles

## Abstract

The longevity of cratons usually implies that the entire cratonic lithosphere remained unchanged over billions of years, which is traditionally attributed to their intrinsically buoyant and strong lithospheric roots. By reviewing relevant studies and recent observational constraints, we show that the present cratonic roots are notably denser than the ambient mantle, with the compositional buoyancy offsetting only one-fifth of the negative thermal buoyancy. In addition, the presence of a weak mid-lithospheric discontinuity could decouple the upper and lower lithosphere upon perturbation, allowing delamination of the lower portion, while most of the delaminated lithosphere would eventually relaminate to the base of the lithosphere after sufficient warming inside the convective mantle. This process generates enduring (>100 Myr) and prominent (>1 km) surface uplifts within continents, a mechanism more compatible with data, especially those reflecting lithospheric deformation, than the model of all continents climbing up a steady region of dynamic uplift. Subsequent lithospheric cooling gradually draws the surface down to below sea level, where the lithospheric mantle density reaches a maximum upon formation of the next supercontinent. We argue that such cratonic deformation has happened repeatedly over supercontinent cycles since the Neoproterozoic and has largely shaped the properties of the present cratonic lithosphere. A few new research directions are also suggested.

## Teaser

Mantle rocks hundreds of kilometers below cratons are long thought to be stable since formation. Recent studies suggest that these rocks experienced repeated deformation and alteration over supercontinent cycles.

## INTRODUCTION

**Figure 1.**
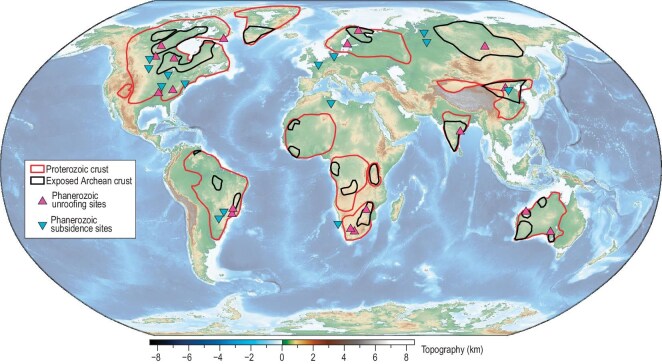
Topography and Precambrian crustal provinces. Both Archean and Proterozoic crusts are generally low and flat, while Phanerozoic crusts have much more diverse topographic features. Locations of major Phanerozoic uplift (magenta triangles) and subsidence (cyan reversed triangles) events overlying present-day surface topography are shown (modified from ref. [26]).

What sets Earth apart from other terrestrial planets is its two distinct types of crusts: oceanic versus continental. While the mafic oceanic crust is considered compositionally similar to that of other rocky planets, the continental crust consists of predominantly felsic-to-intermediate compositions whose density is significantly lower than that of mafic rocks. This unique lithospheric configuration of Earth corroborates the theory of plate tectonics, according to which the dense oceanic crusts frequently recycle back into the convecting mantle through subduction while the buoyant continental crusts avoid being destroyed by passively drifting around the Earth's surface. A closer examination of geological records suggests that continents near plate boundaries are nearly constantly deforming through time. In contrast, the perceived stable and long-lived continents usually refer to their ancient Precambrian cores, cratons, which cover 50%–60% of the total continental area (Fig. 1). Historically, the concept of the craton was defined based on crustal properties by referring to continental regions with ancient (pre-Phanerozoic) and structurally intact sedimentary strata or crystalline basement [1]. In the context of plate tectonics, cratons are further characterized as tectonic units with thick (up to 300 km) mantle roots, as revealed by seismic tomography [2]. This paper revisits the definition of craton by examining the properties and evolution of its lithospheric mantle.

The apparent stability of cratons has been credited to their refractory lithospheric mantle. This refers to not only the density deficit of this mantle layer due to its depletion of iron (ideally following a state of isopycnicity [3,4] where thermal and compositional density effects cancel out), but also to its large mechanical strength and strong crust–mantle coupling due to the depletion of volatiles [5–7], both resulting from high degrees of melt exaction during craton formation [1]. However, the isopycnicity hypothesis has, since this concept was proposed, received mixed responses from both geophysics [8–10] and geodynamics [11–16] communities. The implied temporal stability of the cratonic lithosphere has encountered numerous challenges; for instance, seismology has shown layered lithospheric fabrics [17–20], geology has revealed intracratonic uplift and subsidence [21,22], and geodynamic modeling has suggested lithospheric delamination beneath cratons [23–26]. It is worth noting that it is not only cratons near subduction zones [27,28] and continental rifts [29] that could be severely altered or even destroyed. Those far from active plate boundaries could have also experienced significant changes over time [23,26]. This paper aims to discuss the nature of cratonic lithosphere and the mechanisms governing its secular evolution.

Here, we review the history of our understanding on the structure, density and dynamics of the cratonic crust and its lithospheric mantle, as well as the corresponding implications for craton tectonics, and reveal enormous variations in perspectives over time. We present our most recent interpretations of craton properties and their evolution, which match a variety of observational constraints, with a predictive power much improved compared to previous models. Based on this, we further propose that the cratonic lithospheric mantle has experienced periodic secular deformation, largely following supercontinent cycles, since as early as the Neoproterozoic. We conclude by sharing some thoughts on potential future research directions with regard to further studying the Earth's cratonic lithosphere.

## STRUCTURE AND DYNAMICS OF CRATONIC LITHOSPHERE

In a freely convecting Earth, the long-term stability of a lithosphere ultimately boils down to its buoyancy and high strength relative to surrounding plates. Consequently, the contrasting views on the dynamic nature of cratons stem from inconsistent knowledge on the thickness, composition, density and deformation history of their crust and lithospheric mantle. Here, we present a brief overview of the observations and interpretations of these fundamental lithospheric properties over the past decades.

### Controversies regarding the definition of continental isostasy

**Figure 2.**
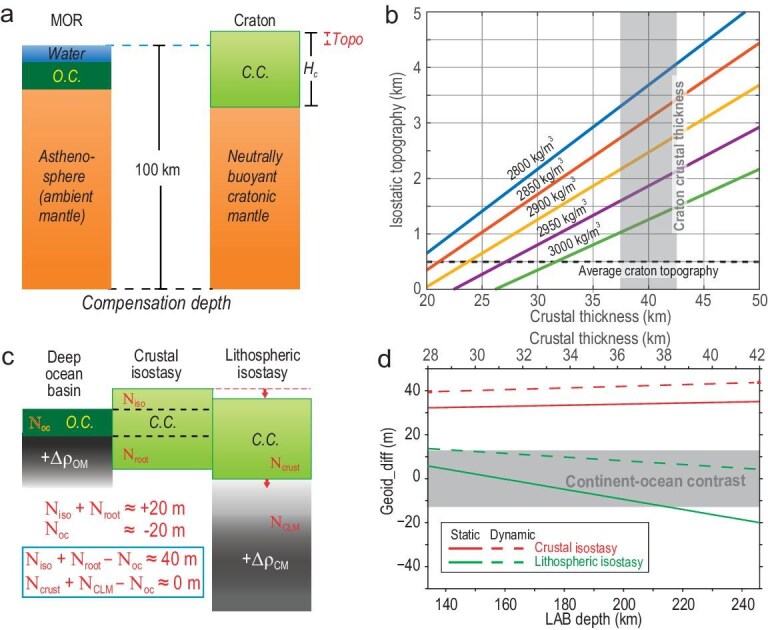
Topography and geoid constraining the isostatic density structure of the cratonic lithosphere. (a) Illustration of the isostatic balance between a mid-ocean ridge and the cratonic interior, assuming neutrally buoyant CLM. (b) Isostatic topography vs. crustal thickness for different crustal density values following the scenario in (a). The vertical gray bar marks the observed thickness range of cratonic crusts. The horizontal dashed line shows the observed mean craton topography. (c) Geoid effects of MOR and cratonic interiors for two isostatic scenarios, one with a neutrally buoyant CLM and another with a dense CLM. (d) Geoid difference between craton and ocean for the two isostatic scenarios in (c).

It is well established that due to the presence of a weak asthenosphere, the force balance of Earth's lithosphere system (both between oceans and continents, and among different types of lithospheric units) largely follows isostasy. Therefore, the properties of the lithosphere can be estimated by relating the thickness and density of its internal layers to surface topography and gravity (or geoid). Since the asthenosphere is an unconfined (with a surface opening like the mid-ocean ridge (MOR)) low-viscosity channel, it cannot sustain long-term lateral pressure gradients, therefore marking an ideal depth for isostatic compensation of the overlying lithosphere. Given that the density profile of an oceanic plate, especially the young portion, is well quantified, one can estimate the buoyancy of a continental plate by ‘weighing’ it against that of the young seafloor, where the simplest configuration is a MOR (Fig. 2a).

However, this intuitive approach is not necessarily a common exercise, even in most recent studies. Instead, researchers evaluating the isostatic balance of continental lithosphere persistently choose different reference topographies (or mass columns) for continents and oceans, rendering their direct comparison unphysical with inconsistent conclusions on the inferred lithospheric density structure. Among the numerous definitions of continental isostasy, we name just a few examples here: (i) freely adjusting continental thickness and density to match topography [9]; (ii) shifting the residual continental topography (real topography subtracted by crustal effects) to avoid abrupt contrasts with that of the ocean basins [13]; (iii) extrapolating measured oceanic residual topography to continental regions following the trend of gravity anomalies [30]; (iv) different choices of a global reference mass column, such as one that matches the globally averaged continental topography [31], one that satisfies the regionally averaged continental topography [32], or adopting the globally averaged zero-elevation continental column [33]. Obviously, there is no way the resulting continental lithosphere structure from these studies could converge. Consequently, estimating the lithospheric buoyancy structure based on this traditional philosophy is unreliable (due to a somewhat arbitrary reference frame for continental isostasy). As shown later, all these earlier studies significantly underestimated the density of the cratonic lithospheric mantle (CLM), since most of them chose the CLM (or part of it) as the reference point, thus eliminating (or hiding) all its intrinsic density anomalies and producing interpretations of craton evolution drastically different from what is presented below. Here, we will adopt the MOR as a globally uniform reference level, based on which we infer the density structure of the continental lithosphere using basic data without adjusting or assuming things (Fig. 2a and b).

**Figure 3.**
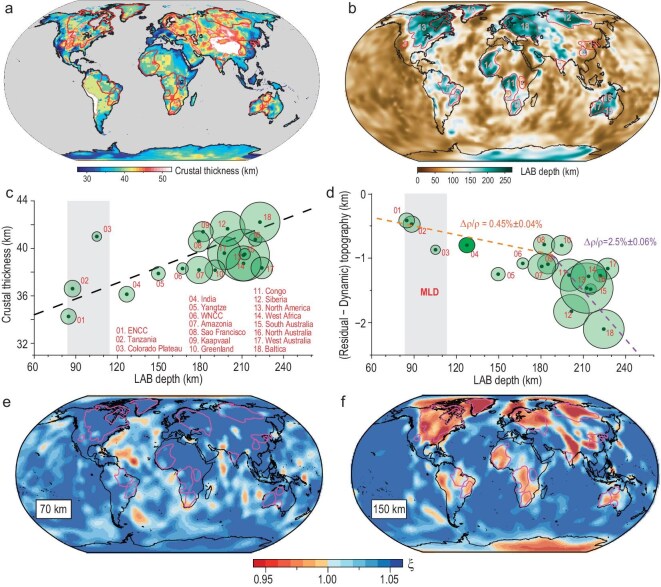
Geophysical characteristics of global cratonic lithosphere. (a) Crustal thickness of continents from CRUST1.0 [36]. (b) LAB depth [26], with the number index of cratons indicated. (c) Correlation of crustal thickness with LAB depth for all cratons. The dashed line is a linear fit for the overall correlation [26]. (d) CLM-induced topography vs. LAB depth, showing the two-layer density structure of CLM [26]. The gray shaded region marks the depth range of MLD. (e, f) Radial anisotropy (ξ = V_SH_/V_SV_) of the CLM at depths of 70 km and 150 km [18], revealing contrasting rock fabric and deformation history above and below the MLD.

Efforts in calculating the density structure of continental lithosphere can be traced back to the 1970s, when the theory of plate tectonics started to gain popularity. However, due to inadequate constraints on the characteristics of continental lithosphere—many modern geophysical and geochemical methods were not yet available—different treatments or assumptions with regard to these properties often resulted in inconsistent interpretations of lithospheric properties and dynamics. Among these treatments, two are the most crucial. The first concerns the thickness and composition of the crust and lithospheric mantle. Traditionally, the crust is considered the primary or only candidate for controlling continental topography and gravity anomalies. The early proposal of isopycnicity, that is, the lithospheric mantle is neutrally buoyant and does not contribute to topographic variations [3,12], further strengthened the belief in pure crustal isostasy for continents. However, because crustal thickness measurements were sparse and inaccurate in the first few decades after the 1970s, geodynamic calculations often adopted different assumptions about Moho depth and/or lithospheric density profiles. One common approach was to use a continent-ocean function to match topography and gravity data, which usually required somewhat arbitrary adjustment in crustal thickness and/or density [9,34,35]. To match these data, these early studies required the continental crust to be <35 km thick, but more recent seismic studies [36,37] consistently reveal much thicker continental crusts, with an average thickness below cratons of ∼40 km (Fig. 3a), thus challenging the validity of these results. It is likely that, because the isostatic contribution (relative to the MOR) of the thick continental crust is so large (Fig. 2), some recent studies chose the continental interior as the reference column [31–33] in order to diminish the residual topography, an exercise that led to considerable debate on the inferred lithospheric density.

The second treatment crucial for understanding lithospheric buoyancy centers on the topographic contribution from non-isostatic mechanisms, whose long-wavelength (>300 km) components originate from the convective mantle, namely dynamic topography [38]. Since isostatic topography reflects the effect of the ‘strong’ outermost shell of the Earth and dynamic topography manifests the effect of the ‘soft’ convective interior of the Earth [39], it is important to revisit the concepts and framework for their respective calculations. As noted above, due to limited knowledge about the deep Earth, the concept of isostasy traditionally only applied to the crust. This perception led many early researchers to consider the topographic contribution from anywhere below the crust as being dynamic in nature [34,40,41], implicitly assuming that the strong lithospheric mantle is convective, a notion that tends to violate the longevity of the continental lithosphere. This is the case in the traditional continent-ocean function, which considers the global mean residual topography as the reference level (a property of a fluid or of dynamic topography whose global mean defines the sea level); in this case, the magnitude of residual topography is very large (>2 km) and the global MOR system is dynamically uplifted by >1 km, inconsistent with the consensus that the MOR should be free of dynamic topography. Furthermore, this makes it practically impossible to evaluate the topographic contribution, thus density structure, of the lithospheric mantle itself, given the large depth range of the mantle and its complex internal density structure, all of which could influence surface topography. On the other hand, some researchers consider the entire lithosphere, as long as it is not subducted or delaminated, as the defining unit of isostasy [10,15,42]. Clearly the latter definition is more intuitive and appropriate for evaluating the nature of cratonic lithosphere and is what we will adopt here. As recently demonstrated, seeing the CLM as part of the isostatic system helps to reduce the magnitude of dynamic topography with the MOR free of dynamic uplift [15].

### Density profile of the cratonic lithosphere

With the above discussions in mind, we revisit the density structure of the continental lithosphere. We first evaluate the traditional model of isopycnicity, which means the lithospheric mantle below a craton has the same density as that of the ambient mantle. In this case, the global compensation depth could be anywhere below the thickest crust, and a nominal value of 100 km is taken here. As Fig. 2a shows, the hydrostatic pressure at this depth, below a MOR, should be equal to that at the same depth below a craton. The densities and thicknesses of all tectonic units in this system are known, including water (1.0 g/cm^3^, 2.5 km), continental crust (2.85 g/cm^3^, 40 km), oceanic crust (3.0 g/cm^3^, 7 km) and ambient mantle (3.3 g/cm^3^, from the base of the crust to 100 km depth). Applying these values to the force balance in Fig. 2a produces a cratonic isostatic topography at 3.1 km above sea level (Fig. 2b). This is significantly higher than the observed craton topography, most of which sits at ∼0.5 km (Fig. 1).

Trying to match the 0.5 km elevation requires abnormally thin (<25 km) crusts or unreasonably high crustal density (>3000 kg/m^3^), a conclusion echoing earlier efforts with limited constraints on crustal properties [9,35]. It is evident that with observed crustal thickness (40 km on average) and plausible mean densities (2800–2900 kg/cm^3^), pure crustal isostasy cannot generate the common craton topography (∼500 m on average, Fig. 2b). This requires the cratonic root to be notably denser than the ambient mantle. Quantitative calculations show that, by further correcting for the effect of dynamic topography, a depth-averaged density anomaly of ∼1% should be present within the present mantle root [15]. This amount of density anomaly is close to (only slightly smaller than) that of an oceanic lithosphere (Fig. 2c). Therefore, the apparent stability of continents should benefit mostly from its buoyant crust. Earth's geoid is another key constraint with regard to inferring the density structure of the Earth. Its greater sensitivity to the depth of mass anomalies could help further pin down the location of the inferred high-density composition within the CLM. Traditionally, the fact that the prominent topographic contrast between continents and oceans is not associated with a similar contrast in the geoid (or gravity) is generally attributed to isostasy by assuming the geoid contribution from the positive topography and that from the crustal root cancel out (Fig. 2c) [4,30]. Based on this assumption, the lack of prominent geoid within cratonic interiors was further translated as reflecting the neutral buoyancy of cratonic lithospheric mantle [8,12]. However, a closer look at this problem indicates that a compensated thick continental crust should still generate an anomalously large positive geoid (0.5 km surface topography corresponds to +20 m of geoid high) within its interior [43], and the same finding also applies to the ocean where a notable (−20 to −40 m) negative geoid is associated with the deep ocean basin [14]. This means that the lack of prominent continent-ocean geoid contrasts (>40 m if continental topography is solely controlled by the crust) goes against pure crustal isostasy (Fig. 2c).

More quantitatively, both static (not considering dynamic adjustment of topography due to mantle flow) and dynamic (with dynamically consistent topography and mantle flow) geoid calculations confirm the above inference (Fig. 2d) [16]. Applying this analysis to global geodynamic models further shows that a denser-than-ambient CLM could effectively eliminate the 40-m continent-ocean geoid contrast (Fig. 2d) that is unavoidable in the case of pure crustal isostasy, a conclusion corroborating the finding based on topography (Fig. 2b). Geoid modeling further pins down the depth range of the CLM density anomaly by requiring the excess density (depth average 0.5%–1.2% denser than the isopycnicity state) to mostly reside in the lower half of the CLM (Fig. 2c and d) [16]. This on one hand means that the CLM has a prominent negative buoyancy similar to (although with a smaller amplitude than) that of an oceanic lithosphere, but on the other hand it contrasts that of an oceanic lithosphere, which has a larger density anomaly within the shallower portion of the lithosphere, due to colder temperatures closer to the surface (Fig. 2c). Here, the existence of a high-density lower CLM (Fig. 2c) represents a result different from all previous studies [26].

A seemingly alternative interpretation of the dense CLM we arrive at is to consider all ocean basins overlaying a 200-km thick mantle layer with a mass deficit of ∼1%; for instance, the oceanic asthenosphere is filled with material more buoyant than both the surrounding continental lithosphere and the mantle underneath it. The relatively simple expression of seafloor bathymetry means that this layer must be laterally uniform to not disrupt the dominant age dependence of bathymetry, a requirement that seems at odds with the laterally variable seismic structure of the sub-ocean mantle [44]. We therefore prefer the existence of a dense CLM below most cratons on the present Earth. In future, many other alternative models may be advanced, but they all need to consider the various geophysical and geological constraints presented in this paper.

### Evolving views on the mechanical strength of cratonic lithosphere

The high viscous strength of the cratonic lithosphere and the strong mechanical coupling between the crust and underlying lithospheric mantle are considered another key reason for the longevity and stability of cratons [6,45]. These mechanical properties are primarily related to the low temperature of the cratonic lithosphere, which has the smallest heat flow values among all tectonic units. In addition, the excessive melt depletion and dehydration of the cratonic roots are thought to further strengthen the CLM, resulting in prominently large viscosity contrasts (more than one order of magnitude) between the lowermost mantle root and the top of the asthenosphere [46–48]. This configuration differs markedly from the gradual viscosity decrease with increasing depth, if only the temperature effect is considered.

Numerical modeling suggests that large-enough rheological contrasts (no less than three orders of magnitude) between the cratonic root and asthenosphere might prevent the cratonic root from being eroded away by the convective mantle for billions of years, even without considering the effects of the chemical buoyancy of the root [45,49]. However, the theoretically calculated viscosity strengthening factor of the cratonic root, due to dehydration based on data of mantle water distribution, ranges from one to four orders of magnitude [50,51], not guaranteeing absolute stability. In addition, the plausible high density of the CLM as discussed above further compromises the effect of high viscosity on the long-term stability of craton roots.

Besides the absolute strength of the craton root, the mechanical coupling among the internal lithospheric layers is also important for its longevity. For example, the craton crust and the lithospheric mantle both have high strength and form strong coupling between them [52] that in turn maintains the long-term stability of the craton lithosphere [6]. In this case, the strong crust contributes to the high strength of the entire lithosphere and prevents the subcrustal mantle detaching [6,52], while the strong lithospheric mantle underneath separates the crust from the convective mantle to protect the crust from being thermally or compositionally altered [27].

However, the cratonic lithosphere bears many structural heterogeneities, as increasingly revealed by observation [2,53,54]. Many of these heterogeneous structures consist of orogenic belts resulting from the long-term deformation and evolution of cratons, such as the repeated amalgamation and accretion of continental blocks. These structural heterogeneities could strongly affect the mechanical strength of the lithosphere by forming weak zones/belts traversing the cratonic platform [55,56], such that these cratonic blocks preferentially deform or separate along these zones during supercontinent evolution [57].

**Figure 4.**
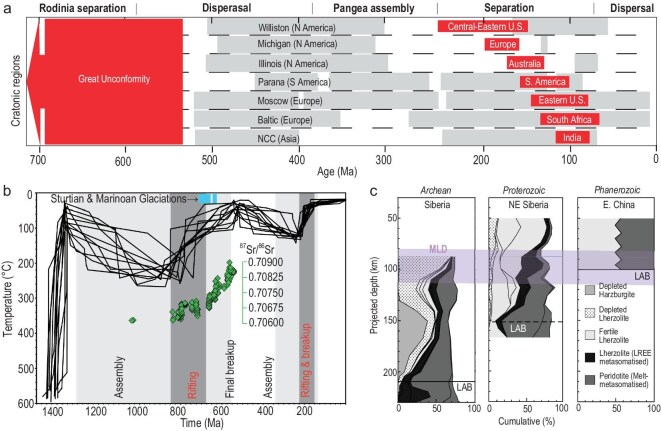
Geological records indicating craton vertical motion and lithospheric mantle composition. (a) Thermochronologically inferred craton uplifts (red shades) and stratigraphically inferred basin subsidence (gray shades) within cratonic basins (from ref. [26]). The uplifts and subsidence phases correlate with separation and assembly (or dispersal) of supercontinents. (b) Burial and unroofing history of Ozark Plateau, in the southern part of the North American craton (from ref. [22]). Note the strong correlation with supercontinent cycles. (c) Petrological structure of CLM below Archean, Proterozoic and Phanerozoic crusts, respectively (modified from ref. [107]).

Besides the vertically oriented orogenic belts that cut through the lithosphere, a recently discovered structural feature below cratons is the mid-lithospheric discontinuity (MLD), which marks the top of a layer with relatively low velocity within the overall high-velocity cratonic lithospheric mantle [53,58]. The depth of this layer (70–120 km) also separates the CLM into an upper and lower sublayer (Figs 3c, 4c). The origin of the MLD remains elusive, with proposed models including accumulations of volatile-rich minerals that are stable at the MLD depths [59–61], a depth of phase change [62], and a transitional depth of seismic anisotropy (Fig. 3e and f) [18,20]. Recent seismic imaging combined with regional tectonic histories suggests that the MLD likely represents a mechanically weak layer that could effectively decouple the upper and lower CLM during craton deformation [23,58]. This inference has found further support from recent geodynamic models [25,63–66].

### Implications regarding composition and stability of cratonic lithosphere

The inferred lithospheric density structures from both topography and the geoid (Fig. 2) directly challenge the traditional isopycnicity hypothesis and the associated tectosphere model. The inferred ∼1% depth-averaged excess density anomaly converts to a thermal anomaly of ∼300$^\circ $C. Taking a nominal cratonic Moho temperature of 500$^\circ $C [67] and LAB (lithosphere–asthenosphere boundary) temperature of 1350$^\circ $C, the depth-averaged temperature anomaly (relative to the asthenosphere) of the lithospheric mantle is 425$^\circ $C, not too far from the above inferred affective thermal anomaly (300$^\circ $C) within the CLM. Since a 425$^\circ $C temperature difference corresponds to a 1.28% density excess, the inferred 1% net density anomaly means only approximately one-fifth of the lithospheric thermal buoyancy is offset by the compositional effect, implying that the cratonic lithospheric mantle is not as refractory as previously thought and high-density components are present within the lower lithosphere. It is possible that these dense materials are not adequately sampled by xenolith data [68], as entraining them in ascending magmas is more difficult than the entrainment of lighter rocks. A possible origin or mechanism for the dense materials may include incomplete draining of ancient mafic crusts [69], post-cratonization metasomatism [68] or mantle refertilization [70]. Furthermore, the dominant presence for these dense materials within the lower lithosphere (Fig. 2c) disfavors the hypothesis of ancient mafic crusts as they tend to stagnate within the upper lithosphere [69]. In any case, the existing petrological models of cratonic lithosphere need to be reevaluated.

Consequently, a high-density CLM would affect the traditional definition of craton stability. We reiterate that the original concept of cratons was mostly based on crustal properties while the role of the mantle root was introduced later as a potential mechanism for the craton's longevity. It is mostly the latter component (the CLM) whose role in the definition will be revised, in addition to the meaning of craton stability. On one hand, the dense CLM helps to draw the craton surface downward to near sea level, thus helping to prevent its crust from being eroded too soon. This is a healthy correction to the traditional tectosphere model, which, without heavy mantle roots, implies all crusts should be unreasonably thin (<30 km) to maintain the observed low elevation (Figs 2b, 3a). On the other hand, gravitational instability within the dense CLM is likely inevitable. However, since most cratonic crusts remained intact since their formation, the implied lithospheric instability may be mostly restricted to the denser lower CLM that may decouple from the overlying upper lithosphere, as recently proposed [23–25]. Indeed, according to these studies, the MLD marks the top of a conditionally weak layer that allows the dense lower lithosphere to detach (delaminate) while the buoyant upper lithosphere remains relatively undeformed.

The above two aspects also suggest a revision to the current understanding of craton evolution. On the crustal side, while it remains true that lateral crustal deformation is negligible, vertical movements due to CLM density variation (such as via delamination) could be significant and may notably reduce crustal thicknesses by removing surface rocks through erosion, which in extreme cases could remove most of the upper crust, as seen in the Canadian shield [1,27]. A corollary of this reasoning is that crustal thickness would be strongly dependent on lithospheric thickness for craton regions, as is consistent with the observation that thinner crusts generally correlate with smaller LAB depths and vice versa (Fig. 3c) [26]. In contrast, this linear correlation should not exist according to the traditional tectosphere model, since neutrally buoyant CLM would not affect topography or crustal thickness.

On the mantle side, the decoupled evolution of the upper vs. lower lithosphere implies that these two layers would have different histories of evolution. If the upper (such as <100 km depth) lithosphere has been stable so as to avoid direct interaction with the asthenosphere, its composition could have remained largely pristine, in contrast to the lower lithosphere that more frequently exchanges materials with the convective mantle. This is consistent with the smaller density anomalies of the upper layer (due to its more refractory nature) as inferred from the topography [15] and geoid [16] and the overall higher density of the layer below the MLD where dense materials seem to concentrate below 180 km depth (Fig. 3d). Another important verification of the decoupled upper-lower lithosphere evolution comes from the patterns of radial seismic anisotropy in global CLMs (Fig. 3e and f), where all CLMs above the MLD display horizontally fast polarizations while that below the MLD display vertically fast polarization. This observation is consistent with the upper CLM retaining its ancient fabric while that of the lower CLM reset by vertical deformation such as delamination and relamination [25]. It is worth noting that such a unique anisotropy characteristic does not fit with lithosphere-scale shortening [54,71] or heat-pipe tectonics [72] proposed for the initial craton formation because neither of them would imply layered lithospheric fabrics.

## SECULAR CRATON DEFORMATION AND EVOLUTION

While geophysical data presented in Figs 2 and 3 provide crucial information on the density structure and plausible geodynamic properties of the CLM, they could not provide accurate information on the CLM's secular evolution. Geological records, among all disciplines, preserve unsurpassable amounts of temporal constraints. Here, we select a few lines of pertinent geological records and discuss their implications for the evolution of the cratonic lithosphere.

### Cratonic topography variation over supercontinent cycles

In an instantaneous force balance, the spatial pattern of surface topography holds key information on the concurrent density structure of the lithosphere. Therefore, changes in cratonic topography over time could reflect secular changes of CLM density structure and thickness, assuming that cratonic crusts remain relatively more stable. One potential complication comes from the underlying convective mantle, where the evolving subduction history will impact the overriding plate through dynamic topography [42]. Understandably, this type of topography is most prominent near the convergent plate boundaries [73,74] and its effect within the continental interior is usually subtle and transient [31,38,75,76]. In the case of abnormally shallow subduction, prominent dynamic topography could temporally propagate further inland, but this process has a characteristic time scale of ∼10 Myr [77,78], much faster than the tempo of isostatic topography changes within continental interiors, which largely accompanied the supercontinent cycle (Fig. 4a).

To comprehend the secular evolution of the cratonic lithosphere itself, we try to avoid subduction-induced topographic variations by focusing on the cratonic interiors. Contrary to the traditional expectation that cratonic platforms should remain stable in surface topography and lack changes in sedimentary records, most cratons have unique characteristics. For example, at present, many cratons are low and flat, like Amazonia, Baltica and North America (Fig. 1), consistent with their long-term tectonic quiescence during which weathering and erosion gradually denudated their surface toward sea level. However, some cratons currently display high elevation and rugged topography, like São Francisco and Kaapvaal (Fig. 1). These geomorphic properties must have been obtained relatively recently, given that the efficient climate effect would have smoothed sharp topographic features relatively fast (during a period much less than a supercontinent cycle) [79,80]. A closer examination reveals that these high topographies were indeed relatively young, with those in Wyoming elevated mostly during the Cenozoic [77,81,82] and those in South America and South Africa developed since the late Mesozoic [23,79,83]. Geophysically, these regions all have relatively thin lithosphere (Fig. 3b), implying lateral lithosphere stretching or loss of dense cratonic roots.

In addition to the current high-elevation cratons that reflect a recent increase in surface topography, many of those that are at normal elevation today also preserve a rich record of past topographic variations (Fig. 4a). For example, the Canadian Shield, the oldest core of North America, once experienced significant surface exhumation likely due to dramatic surface uplifts, during the Neoproterozoic [84], a process that denuded much of its upper crust with its current surface floored by widespread metamorphic lower crust and with a crustal thickness of only 36 km (Fig. 3a). The Neoproterozoic also witnessed significant uplift and erosion within other cratons, a tectonic event that was nearly global in scale and last an enormous period of time (with a sedimentary hiatus of more than 1 billion years over many cratons) such that the missing sedimentary records constitute the enigmatic ‘Great Unconformity’ (Fig. 4a) [84,85]. This largely accompanies the separation of supercontinent Rodinia [26]. Another similar but less significant exhumation event occurred during the Mesozoic as supercontinent Pangea separated. The records are mostly distributed within cratons around the Atlantic and Indian oceans, where supercontinent Pangea used to reside (Fig. 4a) [26]; this Mesozoic unroofing event may be considered a secondary global unconformity.

Besides these prominent global uplift events of cratons, the distributed intracratonic basins also preserve a remarkable record of their subsidence history (Fig. 4a) [21,86,87], where the sediment thickness locally exceeds 10 km [88]. Interestingly, these basins formed mostly during the dispersal of continents [21,57] and the associated subsidence history is also consistently later than the major exhumation events during supercontinent separation (Fig. 4a), implying that these vertical oscillations of cratons may share a common mechanism. Indeed, several detailed low-temperature thermochronology studies revealed repeated (every few hundred million years) burial and unroofing of 5–7 km crustal rocks within the North America craton [22,85,89,90], closely following the assembly and break-up of Rodinia and Pangea (Fig. 4b). This history of successive vertical movements embodies the global observation among many cratons (Fig. 4a) [91].

### Mechanisms of CLM deformation

To comprehend the plausible geodynamic processes behind preserved craton records, we will analyze these data constraints in the light of CLM evolution. In practice, we focus on interrogating the topographic records and associated geophysical properties.

**Figure 5.**
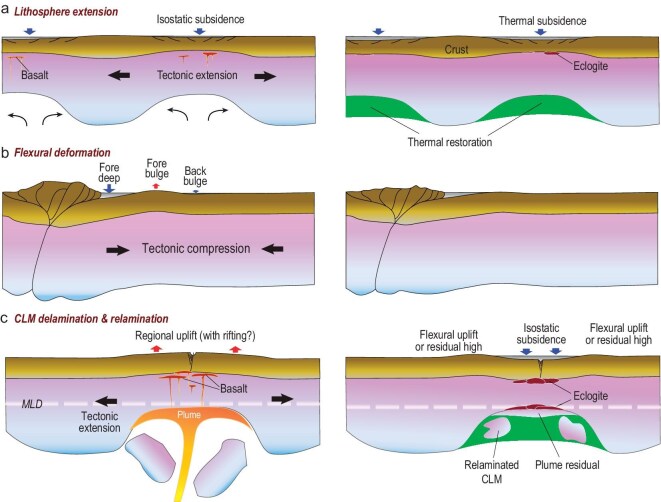
Three representative models for cratonic vertical motion (modified from ref. [39]). (a) Lithosphere extension initially thins the crust and lithospheric mantle, causing surface subsidence (left). Subsequent cooling and regrowth of the thinned lithospheric mantle generates further surface subsidence (right). (b) Lithosphere flexure from orogenic loading causes alternating topographic variations, with the magnitude of main vertical-motion structures decreasing landward (left). The topography remains largely unchanged after the orogeny ends (right). (c) Delamination of the dense lower CLM due to sub-lithospheric perturbations like plumes causes prominent surface uplift, with potential crustal rifting and magmatic underplating (left). Subsequent relamination of the foundered CLM segments and cooling stabilizes the lithosphere, leading to long-term subsidence of the craton surface (right).

The identified topographic history of global cratons (Fig. 4a and b) clearly challenges the traditional wisdom of craton stability in that their surfaces went up and down over supercontinent cycles, with the amount (2–3 km as implied by Fig. 4b) of surface vertical motion far exceeding that of global sea level change (on the order of 100 m). However, the mechanical origin of these widespread (>100 km across) and enduring (>100 million years) uplifts and subsidence features within the stable continental interior remains heavily debated (Fig. 5). The published mechanisms for widespread continental vertical motion could be categorized as dynamic topography or flexural-isostatic topography.

Among the dynamic topography models, one intuitive explanation is that all continents cruised over spatially fixed mantle upwellings such as the two large low-shear wave velocity provinces (LLSVPs) at a somewhat synchronized pace, and by doing so the continents experienced dynamic uplift and subsidence, similar to that proposed for the post-Jurassic vertical motion history of southern Africa [92]. However, this hypothesis seems to have multiple flaws when applied to supercontinent cycles. First of all, the multi-100 Myr timescale of interest seems to fall beyond that of dynamic topography, whose cyclic behaviors due to subduction usually operate on a sub-100 Myr scale [38,93]. As far as the LLSVPs are concerned, recent studies suggest that their spatial fixity might not be able to sustain for more than 200 Myr since the configuration of global subduction zones has been constantly evolving [94,95].

Second, the amplitude of very-long-wavelength (>1000 km) dynamic topography is debated, and it may be anticipated that this amplitude scales inversely with the stability of LLSVPs, with a smaller amplitude corresponding to weaker convection (thus greater LLSVP stability). Following this reasoning, the ultra-stable LLSVPs (>500 Myr) [96] may feature only mild (i.e. 100 m) dynamic uplift that falls short of explaining the much greater (multi-km) surface uplift/subsidence as suggested by geological records (Fig. 4b). Indeed, the compatibility of LLSVPs’ long-term stability [97,98] and the operation of mobile plates over supercontinent cycles [95] represents an outstanding question that requires more future research.

**Figure 6.**
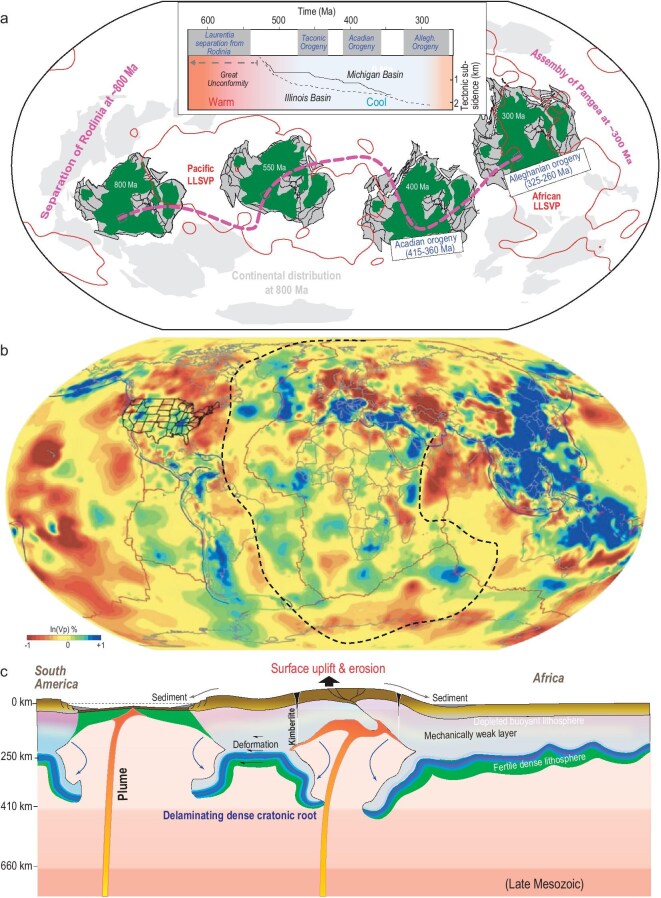
Craton evolution and CLM delamination. (a) Reconstructed location of North America during the transition from Rodinia to Pangea, where light-gray areas represent continents and green areas cratons [118]. The inset plot shows the corresponding North America topography evolution, including two unconformities, two cratonic basins [119] and three orogenic events. (b) Present-day observed fast anomalies, likely representing delaminated CLM, near the base (635 km depth) of the mantle transition zone below the Atlantic Ocean and Indian Ocean, where the dashed black line marks the spatial extent of Pangea around 300 Ma (modified from ref. [120]). (c) A scenario of CLM delamination during the separation of Pangea within the southern Atlantic [23].

**Figure 7.**
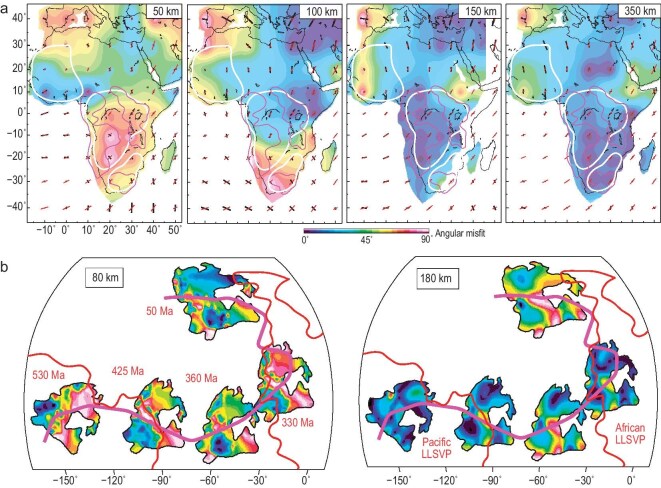
CLM deformation history recorded in azimuthal seismic anisotropy. (a) Correlation of observed African CLM azimuthal anisotropy (black bars) with its Cenozoic plate motion (red bars) at four different depths. The background colors represent the differences (angular misfit) between the two orientations (from ref. [23]). (b) Same as (a) but for North America. A comparison of five different times during the Phanerozoic at two different mantle depths is shown (from ref. [26]).

More critically, as discussed later, these dynamic topography models cannot explain the various lines of lithospheric deformation signals (Figs 3e and f, 4c, 5c, 6, 7), which clearly favor internal deformation of the cratonic lithosphere as the main driving mechanism. Hereafter, we will focus on discussing the lithosphere deformation models.

The flexure-isostasy models largely fall into three categories: (i) the **lithosphere extension model** attributed these subsidence records to crustal or lithospheric stretching and subsequent cooling (Fig. 5a) [21]; (ii) the **flexure model** proposed that these inland basins and abutting topographic highs resulted from tectonic compression or loading from nearby convergent boundaries as back bulge subsidence and fore bulge uplift, respectively (Fig. 5b) [99,100]; and (iii) the **lithosphere delamination/relamination model** argued that alteration of the lower CLM by underlying mantle dynamics such as plumes caused the cratonic surface to go up and down (Fig. 5c) [23,26]. To further evaluate these proposed models, we refer to the geophysical properties of the cratonic lithosphere. As a result of the Neoproterozoic and Mesozoic global uplift events (Fig. 4a and b), many cratons were severely eroded (e.g. North America), and those suffering severe Mesozoic exhumation are still significantly elevated today (e.g. São Francisco, Kaapvaal). A notable consequence is their relatively thin crust (Fig. 3a) and exposure of ancient basement rocks [26]. The fact that high elevation occurs over thin crusts along the southern Atlantic margin in Brazil and Africa disfavors the extension (or crustal isostasy) model which suggests the opposite relationship (Fig. 5a). This conclusion is reinforced by two additional observations: (i) the Canadian Shield (northern part of North America) has coexisting thin crust (Fig. 3a) and thick mantle root, inconsistent with lithospheric extension (Fig. 3b), and (ii) the crust of major cratonic basins (Michigan, Illinois, Siberia, Williston) has large (>40 km) thickness [36,37,101–104]. The flexure model, on the other hand, predicts that prominent vertical motion should occur near convergent boundaries where orogeny occurred and that these topographic signals would remain largely unchanged after the cessation of orogenic processes. However, these predictions are inconsistent with the fact that many cratonic basins are distant from (>500 km) plate boundaries (Fig. 1) and that the onset of orogeny seems to correlate with a slowdown of sedimentation or even basin inversion (hiatus) while the basins subsided more rapidly during orogenic quiescence (Fig. 6a) [105]. Therefore, neither the extension model nor the flexure model represents the main mechanism for the observed cratonic vertical motions.

In comparison, the recently proposed lithosphere delamination/relamination model better matches the geophysical properties. For example, the removal of dense CLM would cause prominent surface uplift, a process that does not require crustal thickening as isostasy does, but instead implies crustal thinning due to surface erosion. In this model, the thickness of the CLM may vary indefinitely, depending on the evolution stage of the delaminated CLM: the lithosphere that experienced delamination during Pangea separation (Fig. 6b and c), such as along the southern Atlantic margin, is still thin since the removed CLM is still within the convective mantle [23,25]; in contrast, cratonic lithosphere that was deformed mostly during Rodinia separation, such as North America, would appear thicker given that the earlier perturbed but not fully detached CLM should have returned back to the base of the craton [25,26] and/or the missing CLM has thermally restored [23]. These geophysical implications are mostly consistent with the present crustal and CLM configuration (Fig. 3b). Consequently, we suggest the delamination/relamination model (Fig. 5c) represents a more plausible mechanism for the observed cratonic records (Figs 1, 3, 4).

An additional check of the three scenario models (Fig. 5) comes from the deep mantle. Both the extension model and the flexure model imply a minimum amount of lithospheric mass loss to the convective mantle. The delamination/relamination model, on the other hand, suggests significant amounts of mass exchange between the lithosphere and underlying mantle. Overall, this seems at odds with the conventional wisdom that cratonic lithosphere would have been exempt from mass exchange with the underlying mantle after their formation [4,106], with the rare exception being that the CLM might be subsequently metasomatized [1,107]. What the delamination model suggests is much more than gradual metasomatic reactions. Instead, large chunks of CLM could detach from the overlying lithosphere and enter the deep mantle (Fig. 6c). This process can not only significantly alter the thermal and compositional structure of the lithosphere while raising surface topography, the delaminated CLM segments may also cool down the ambient mantle and change its composition.

An independent testimony of this scenario comes from seismic tomography, where widespread fast seismic anomalies are imaged at the bottom of the mantle transition zone below most of the Atlantic and Indian oceans, a region largely corresponding to where the last supercontinent Pangea was situated (Fig. 6b). Paleoarc reconstructions suggest that there was no circum-Pangea subduction in this region during 300–200 Ma, when the supercontinent was intact [108]. So, these upper-mantle anomalies should not represent oceanic slabs from then. Neither could they be slabs from later times because all younger subduction zones are beyond the region and each of them link to deep slabs clearly not connected with these anomalies (Fig. 6b). Alternatively, these could be interpreted as delaminated CLM from the underside of Pangea, where the lateral location of these anomalies implies that they separated from the overlying lithosphere before Pangea drifted apart, a scenario consistent with the recent suggestion that CLM delamination occurred mostly during supercontinent separation [23,26].

Another salient aspect of the third craton deformation model is the process of CLM relamination. This is contingent on the condition that the CLM possesses a certain degree of compositional buoyancy such that the delaminated CLM upon reaching the ambient mantle temperature would rise back toward the surface. Although exotic, this scenario is supported by both numerical simulation [23] and ancient CLM material within young seafloors [109,110]. Indeed, relamination represents a necessary step to conserve the lithospheric mass after CLM delamination. Another process that increases CLM mass is melt residual that accumulates at the base of the temporally thinned lithosphere (Fig. 5c), a process sometimes called recratonization [111].

### Periodic instability and long-term evolution of cratonic lithosphere

Understanding the tempo and style of long-term CLM evolution requires knowledge about its tectonic context. Surface records show that major cratonic events are closely associated with the pace of supercontinent cycles, such that dramatic surface uplift associated with CLM delamination occurred during supercontinent separation while basin subsidence associated with CLM cooling and restoration was sustained during the dispersal of continents (Fig. 4). Extending this observation into the deep mantle, the geographic proximity of the supercontinent to the LLSVPs seems to play a crucial role, as plumes rising above the LLSVP likely triggered CLM delamination below the supercontinent (Fig. 6). Here we attempt to analyze the underlying dynamic links among these tectonic components.

At the center of this logic is the dynamic trigger of CLM delamination. The observation that most cratons overlie thick CLM suggests that delamination should not occur too often or randomly. Instead, a tectonically defined mechanism is needed. Since these features are far from subduction zones, mantle plumes become an ideal alternative. Taking North America as an example, its two prominent uplift events (>600 Ma and ∼250 Ma, see Figs 4b, 6a) happened when the craton overlay the Pacific LLSVP and African LLSVP, respectively (Fig. 6a). Since LLSVPs are usually considered the source region of mantle plumes, this correlation supports plumes as a key triggering mechanism for CLM delamination, a conclusion further confirmed by the Mesozoic locations of hotspots relative to disturbed south Atlantic cratons during the separation of Pangea [23].

Given the warming effect of the ambient mantle and the refractory (although less pronounced than traditionally thought) nature of the CLM, most delaminated lithospheric material would eventually return to the base of the lithosphere through the process of relamination (Fig. 5c). Numerical calculation suggests that this process will finish within ∼100 Myr of the initial delamination [25]. It is worth noting that the surface topography will remain high from the stage of delamination to post-relamination, lasting for >100 Myr [25], consistent with the duration of observed global unconformities (Fig. 4) and much longer than that of dynamic topography [38]; it is the subsequent cooling of the lithosphere that gradually draws down the topography from elevated plateaus to eventually deep basins [25,26]. Besides, secular metasomatism, as widely observed within the present cratonic lithosphere (Fig. 4c) [107], could also increase CLM density to cause further subsidence [70]. This trend may continue until the densified CLM experiences another instability and delaminates again, a scenario most likely to occur during the subsequent supercontinent cycle with mantle plumes forming from below. This periodic instability and restoration are reproduced in numerical models [26], supporting its feasibility.

One important consequence of the periodic CLM deformation described above is that the internal structure and rock fabric of the lithosphere would be permanently reshaped. The expectation that the upper CLM remains largely intact while the lower CLM periodically peels off and regrows (Fig. 6c) further suggests the existence of vertical stratification or contrasts of their respective lithospheric fabric. Indeed, examination of seismic radial anisotropy below cratons reveals that the lower CLM has nearly opposite orientation from that of the upper CLM (Fig. 3c), thus confirming the vertically stratified CLM fabrics. This striking observation suggests that the upper and lower cratonic lithosphere likely have decoupled deformation histories. Furthermore, the fast V_SV_ polarization within the lower CLM supports the delamination/relamination-dominated style of vertical deformation of this layer, thus reinforcing our newly proposed craton evolution model (Fig. 6c).

While radial anisotropy delineates the contrasting deformation styles in the vertical vs. horizontal directions, azimuthal anisotropy may record the effect of past plate motion, which in the context of quantitative plate reconstruction can provide a tighter constraint on the timing of CLM deformation. Comparison of lithospheric azimuthal anisotropy with past plate motion directions for cratons that have experienced periodic CLM deformation readily validates the above prediction: the lower CLM fabric preserves the plate motion-induced shear deformation from the time when the craton was disturbed. For example, south-central Africa experienced delamination during the late Mesozoic-early Cenozoic Pangea separation, and the lower CLM fabric of these affected cratonic regions (magenta contours in Fig. 7a) closely follows the Cenozoic plate motion of Africa when its lithospheric structure restabilized (Fig. 7a). Similarly, North America experienced great disturbance during the Neoproterozoic Rodinia separation, and its lower CLM fabric registered the Paleozoic plate motion as the lithosphere restored (Fig. 7b). It is also important to emphasize that both these plates’ upper CLM remained intact during these dynamic perturbations, as reflected in the observation that their corresponding fabric remains unaltered, i.e. not correlating with past plate motions.

**Figure 8.**
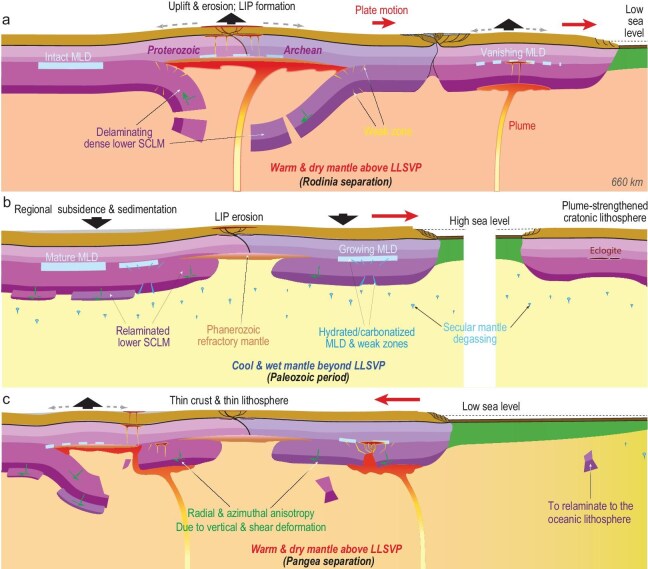
Illustration of the plausible secular evolution of cratons (from ref. [26]). (a) Primed CLM undergoes delamination when perturbed by underlying mantle plumes during supercontinent Rodinia separation. (b) Restoration of the perturbed CLM after relamination, when lithospheric cooling and metasomatism regain pre-delamination conditions. (c) Repeat of the delamination process during the separation of supercontinent Pangea.

Based on a comprehensive analysis of geological records (Figs 1, 4) and geophysical properties (Figs 1, 3), as well as plate reconstructions and deep mantle structures (Fig. 7), we conclude that most cratonic crusts and CLM should have experienced periodic yet differential deformation largely following the pace of supercontinent cycles, following our recent study (Fig. 8) [26]. In this newly proposed craton evolution model, the interaction between the LLSVP-generated plumes and the overriding dense CLM determines the tempo of craton deformation and restoration, with several key stages: (i) supercontinent formation represents the time when most CLMs gain enough negative buoyancy through long-term cooling and metasomatism, and they also develop a mature MLD through volatile accumulation [112] and/or carbonate replenishment [113]; with these conditions, the CLM is primed for subsequent delamination when dynamic perturbations arise. (ii) Mantle plumes represent a major triggering mechanism for primed CLM to undergo delamination, forming various types of unstable structures like fragmentation, peeling off or detachment (Fig. 8); after warming up inside the deep mantle, these foundered CLM segments will become more buoyant than the ambient mantle and eventually ascend to the base of the lithosphere through relamination, during which some may return to their original locations and some could miss the target by forming allochthonous mantle structures. (iii) These returned buoyant CLMs will remain stable for hundreds of millions of years while gradually cooling down to gain their density and regrow the MLD, a process that may be sustained during formation of the next supercontinent, after which the above steps would repeat.

In response to the periodic craton deformation, the crust remains mechanically stable but its long-term vertical movements accompanying the evolving density of the CLM and underlying convective mantle should have shaped the presently observed crustal thickness variation through cumulative exhumation and sedimentation. The lower CLM, whose primed state has a higher density than the upper CLM, retains most of its mass since the up and down motion within the convective mantle mostly changes the thermal buoyancy of the CLM. In some cases, part of the dense materials may get lost as the delaminated CLM may shed some high-density portions into the convective mantle before it can eventually relaminate [25]. Subsequently, these compositional heterogeneities may reaccumulate during the phase of lithosphere restoration, such as through metasomatism (Fig. 8b), a scenario supported by the presence of metasomatized rocks predominantly below the MLD within Precambrian CLM (Fig. 4c). It is worth emphasizing that the upper CLM, during most of these events, would remain mechanically intact due to its prominent compositional buoyancy (Figs 2c and d, 3d, 4c) and large mechanical strength [55], so as to protect the overlying cratonic crust from suffering deformation. A corollary from this reasoning is that some cratons that got severely deformed or even destroyed, like the Wyoming Craton and North China Craton, should have been operated by mechanisms more disruptive than plumes, such as low angle subduction [114–117].

## DISCUSSION AND OUTLOOK

By reviewing research from the past few decades on the structure and buoyancy of cratonic lithosphere, we arrive at a conclusion that the CLM below most cratons is significantly denser than previously thought. This finding stems out of one critical realization: one could accurately measure the density structure of cratonic lithosphere only by placing the reference point beyond the continents. This is because any part of a continental interior is underlain by some lithospheric mantle whose density effect will be zeroed when acting as a reference point, and the results based on this reference only reflect relative internal density variation instead of the absolute density of the CLM. We suggest that the MOR represents the most appropriate reference for all cratons. However, it is still necessary to fully comprehend other relevant studies using different reference frames, as they all present new knowledge to some extent, but a more detailed discussion of them besides what is presented in this review is unfortunately beyond the limit of this review.

Due to the prevalence of the isopycnicity concept [3,4], many observational records pertinent to craton secular evolution have been either de-emphasized or neglected, including the various lines of supercontinent-accompanying geological records on periodic continental vertical motion (Fig. 4a and b) and many geophysical properties of the craton crusts and CLM that deviate from predictions based on the traditional wisdom (Figs 3, 6). Attributing these records to a manifestation of the temporal evolution of the CLM provides many new perspectives on the understanding of cratons. To name a few, the concept of continental isostasy should be extended to include the entire lithosphere instead of only the crust. Regarding the depth range along which CLM delamination tends to occur, the MLD is preferred over the Moho, likely because the former represents a depth where weak minerals prefer to accumulate [59–61]. Delamination along the MLD is unlikely to further damage the upper CLM since the removal of the lower CLM is spontaneous, with a geologically brief period that exposes or alters the upper lithosphere. If by any means the CLM is mobilized along the Moho, the entire cratonic lithosphere will be destroyed, as could happen during low-angle subduction where strong mechanical interactions between the two plates could destroy the overriding CLM [114,117].

While our proposed model of periodic CLM delamination and destabilization could reconcile multiple independent observational constraints, there are still many aspects that need to be further evaluated or quantified, such as the relationship between the present crustal-lithospheric thicknesses and past CLM evolution, the detailed mechanism for reshaping lithospheric anisotropy, and the role of non-isostatic topography within the geological records of surface vertical motion.

Our hypothesis of a dense CLM and its periodic deformation raises many new questions and presents fertile research directions. Therefore, we suggest that future work should pay attention to both further evaluating our proposal and exploring new frontiers along its trajectory. Among these, important areas for exploration include:

- Dynamic effects of an evolving CLM on the histories of Phanerozoic tectonics, sea level, climate and environment.
- The origin and evolution of the CLM structure and composition since the time of craton formation.
- Implications for early Earth processes, including emergence of crust and lithosphere, secular transitions in the dominant tectonic mode, and onset and maintenance of plate tectonics.
